# Cardiotoxic drugs Herceptin and doxorubicin inhibit cardiac microvascular endothelial cell barrier formation resulting in increased drug permeability

**DOI:** 10.1242/bio.020362

**Published:** 2016-08-19

**Authors:** Emma L. Wilkinson, James E. Sidaway, Michael J. Cross

**Affiliations:** 1 MRC Centre for Drug Safety Science, Department of Molecular and Clinical Pharmacology, Sherrington Building, Ashton Street, The University of Liverpool, Liverpool, L69 3GE, UK; 2 Molecular Toxicology, AstraZeneca, Alderley Park, Cheshire, SK10 4TG, UK

**Keywords:** Cardiotoxicity, Cardiac permeability, Anthracycline, Herceptin, Endothelial

## Abstract

Cardiotoxicity induced by anti-cancer therapeutics is a severe, and potentially fatal, adverse reaction of the heart in response to certain drugs. Current *in vitro* approaches to assess cardiotoxicity have focused on analysing cardiomyocytes. More recently it has become apparent that non-cardiomyocyte cells of the heart can potentially contribute to cardiotoxicity. Herceptin and doxorubicin are known to induce cardiotoxicity in the clinic. The effect of these drugs on the endothelial tight junction barrier was tested by analysing tight junction formation and zona occludens-1 (ZO-1) levels, revealing that Herceptin and doxorubicin are able to induce barrier perturbment and decrease barrier function in human cardiac microvascular endothelial cells (HCMECs) leading to increased permeability. Herceptin treatment had no effect on the tight junction barrier function in human dermal and human brain microvascular endothelial cells. HCMECs showed detectable levels of HER2 compared with the other endothelial cells suggesting that Herceptin binding to HER2 in these cells may interfere with tight junction formation. Our data suggests that doxorubicin and Herceptin can affect tight junction formation in the cardiac microvasculature leading to increased drug permeability and adverse effects on the cardiac myocytes.

## Abstract (precis)

**Summary:** The anti-cancer drugs doxorubicin and Herceptin can disrupt tight junction formation in cardiac microvascular endothelial cells resulting in increased permeability which could potentially contribute to drug-induced cardiotoxicity.

## INTRODUCTION

Cardiotoxicity is defined as a severe and potentially fatal adverse cardiovascular event in response to certain drugs and is responsible for the majority of drug development terminations/withdrawals at the pre-clinical and post-approval stage over the last 10 years (Cross et al., 2015; Laverty et al., 2011). Drug induced cardiotoxicity can result in both functional effects such as arrhythmia and acute alteration of the contractile function (inotropy) of the heart, and morphological (structural) damage to the myocardium (Cross et al., 2015).

The cardiac myocardium is composed of cardiomyocytes, which constitute approximately 30% of the total cells, and non-myocytes (ﬁbroblasts, endothelial cells), which constitute approximately 70% of the total cells (Brutsaert, 2003). Cardiomyocytes generate the contractile force while ﬁbroblasts secrete extracellular matrix and paracrine factors (Porter and Turner, 2009; Ravenscroft et al., 2016). Endothelial cells line the coronary vasculature and form a barrier which regulates the movement of oxygen, free fatty acids and xenobiotics from the circulation (Brutsaert, 2003; Tirziu et al., 2010). Paracellular permeability is regulated by endothelial cell-cell junctions. Adherens junctions initiate cell-to-cell contacts and promote their maturation and maintenance, while tight junctions regulate the passage of ions and solutes through the paracellular route (Bazzoni and Dejana, 2004; González-Mariscal et al., 2005, 2008).

The anthracycline doxorubicin is used to treat a variety of cancers such as leukaemia, breast and ovarian cancer (Sartiano et al., 1979). Anthracyclines produce dose-related cardiac dysfunction, deﬁned as type I cardiotoxicity (Ewer and Lippman, 2005), characterised by cardiomyocyte ultrastructural abnormalities (vacuoles, myoﬁbrillar disarray and necrosis), and contractile abnormalities (dilated cardiomyopathy) resulting in reduced left ventricular ejection fraction (LVEF) and heart failure (Billingham et al., 1978). Some elements are initially reversible, but over time the burden of ﬁbrosis and myocyte loss to apoptosis renders the dysfunction irreversible.

Herceptin (trastuzumab) is a humanised monoclonal antibody that specifically targets HER2 (EGFR-2/ERBB2) (Walshe et al., 2006), which is overexpressed in approximately 20% of breast cancers (Baselga et al., 1998). Despite its clinical efficacy, Herceptin has been demonstrated to lead to decreased LVEF in some patients and is classified as type II reversible cardiotoxicity (Lemmens et al., 2007; Sandoo et al., 2014). The mechanism of cardiotoxicity has been attributed to blocking normal HER2 function in cardiomyocytes (Force et al., 2007). Gene ablation of *Her2* in mice leads to embryonic lethality at embryonic day (E)9.5-10.5 due to trabeculae malformation in the heart (Harari and Yarden, 2000). HER2 can heterodimerise with HER3 (EGFR3) and HER4 (EGFR4) following agonist stimulation with neuregulins, which activates an intracellular signalling cascade in cardiomyocytes leading to cell survival (Creedon et al., 2014). The importance of HER2 function in cardiomyocytes is highlighted by the fact that cardiotoxicity of doxorubicin is aggravated by co-administration of Herceptin, which has led to the sequential administration of these drugs in patients to reduce the severity of cardiovascular toxicity (Gianni et al., 2007).

Whilst the majority of studies analysing the molecular mechanism of cardiotoxicity have focused on effects on cardiomyocytes, there is a growing awareness that cardiotoxic anti-cancer drugs can also adversely affect cardiac vascular function (Chintalgattu et al., 2013; Chiusa et al., 2012). Tubulin binding drugs, such as vincristine, have been shown to adversely affect rat cardiac microvascular endothelial cells (Mikaelian et al., 2010), while doxorubicin has recently been shown to affect VEGF signalling in rat cardiac microvascular endothelial cells (Chiusa et al., 2012). We were interested in the possibility that cardiotoxic drugs such as Herceptin and doxorubicin may directly affect cardiac endothelial cell function. Using a number of *in vitro* methods we showed that doxorubicin and Herceptin can affect cardiac microvascular endothelial cell barrier function leading to increased drug permeability. These data suggest that cardiac microvascular injury may be an initiating and contributory event in drug-induced cardiotoxicity.

## RESULTS

### Herceptin and doxorubicin affect tight junction formation and increase permeability in cardiac microvascular endothelial cells

In order for chemotherapy to be effective, the drug must gain access to the tumour from the microvascular capillary bed. The tumour vasculature is relatively leaky due to aberrant angiogenesis allowing effective delivery of chemotherapy (Chung et al., 2010). In contrast, the blood-brain barrier (BBB) presents a relatively impermeable barrier to the delivery of chemotherapy such as doxorubicin and Herceptin to target brain metastasis (Deeken and Loscher, 2007). We were interested in determining the effect of doxorubicin and Herceptin on microvascular permeability using human microvascular endothelial cells from different anatomical locations. We utilised human dermal microvascular endothelial cells (HDMECs), human cardiac microvascular endothelial cells (HCMECs) and human brain microvascular endothelial cells (HBMECs).

**Fig. 1.**
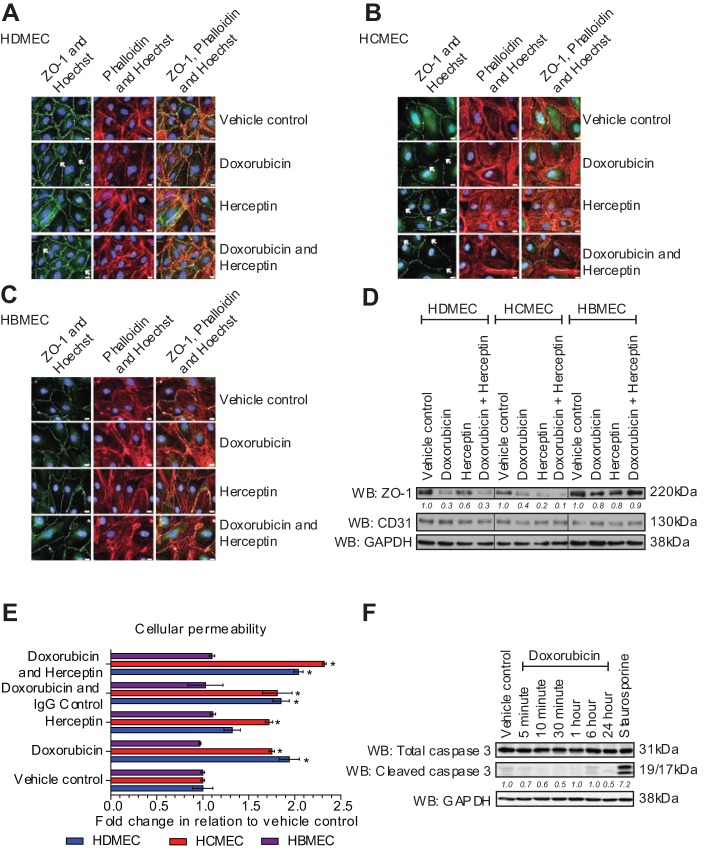
**Effects on the tight junction barrier and permeability following doxorubicin and Herceptin treatment.** Immunofluorescence imaging of endothelial tight junctions (ZO-1, green), actin stress fibres (phalloidin, red) and nuclei (Hoechst, blue) following treatment with doxorubicin 0.1 µM and/or Herceptin 10 µg/ml for 6 h. (A) HDMECs, (B) HCMECs and (C) HBMECs; arrows indicate barrier perturbment. Scale bars:10 μm. Results are from 1 experiment representative of 3. (D) Western blot of ZO-1, CD31 (PECAM-1) and GAPDH levels in HDMECs, HCMECs and HBMECs following treatment with doxorubicin 0.1 µM and/or Herceptin 10 µg/ml for 6 h. Level of ZO-1 is quantified relative to vehicle control level for each cell type. (E) Permeability of 4 kDa dextran-FITC through an endothelial monolayer on inserts, (*n*=4), mean±s.d. *P≤0.05 compared to vehicle control, one-way ANOVA, SPSS. (F) Western blot analysis of cleaved caspase 3 in HCMEC treated with 0.1 μM doxorubicin for different time periods. Staurosporine (50 nM) was added for 6 h as a positive control for apoptosis.

The tight junction barrier was assessed by immunofluorescence staining of the tight junction protein zona occludens-1 (ZO-1). The data presented in Fig. 1A-C demonstrate that doxorubicin induced tight junction barrier perturbment in HDMECs and HCMECs, but not HBMECs. This is in agreement with the fact that doxorubicin does not effectively penetrate the BBB in patients (Blasberg and Groothuis, 1986). Herceptin treatment caused tight junction barrier perturbment in HCMECs only. This suggests that cardiac endothelial cells are more susceptible to Herceptin than endothelial cells from other anatomical locations. Analysis of the level of ZO-1 by western blotting revealed that doxorubicin reduced the protein level in HDMECs and HCMECs. Herceptin had a slight effect in reducing ZO-1 levels in HDMECs but a profound effect in reducing ZO-1 levels in HCMECs, an effect augmented slightly by co-addition of doxorubicin (Fig. 1D). Analysis of CD31 (PECAM-1), a transmembrane glycoprotein involved in cell adhesion and constitutively expressed on endothelial cells (Privratsky et al., 2011), revealed that drug treatment did not affect expression of this protein (Fig. 1D).

To assess the potential physiological relevance of drug-induced tight junction barrier perturbment, an *in vitro* assay was performed to measure permeability using FITC-labelled dextran. Cells were treated with doxorubicin and Herceptin alone or in combination, before the flow of fluorescent dextran through the monolayer was measured (Fig. 1E). This data shows that doxorubicin significantly increased permeability in HDMECs and HCMECs but not in HBMECs. In agreement with effects on ZO-1, Herceptin increased permeability in only the HCMECs, an effect that was increased in the presence of doxorubicin. In order to preclude potential apoptotic effects with doxorubicin leading to barrier perturbment we analysed the level of cleaved caspase 3 following doxorubicin treatment. Significant apoptosis was not observed in the HCMECs with 0.1 μM doxorubicin over a 24 h time period (Fig. 1F).

### Analysis of transporter expression and doxorubicin accumulation in different endothelial cells

**Fig. 2.**
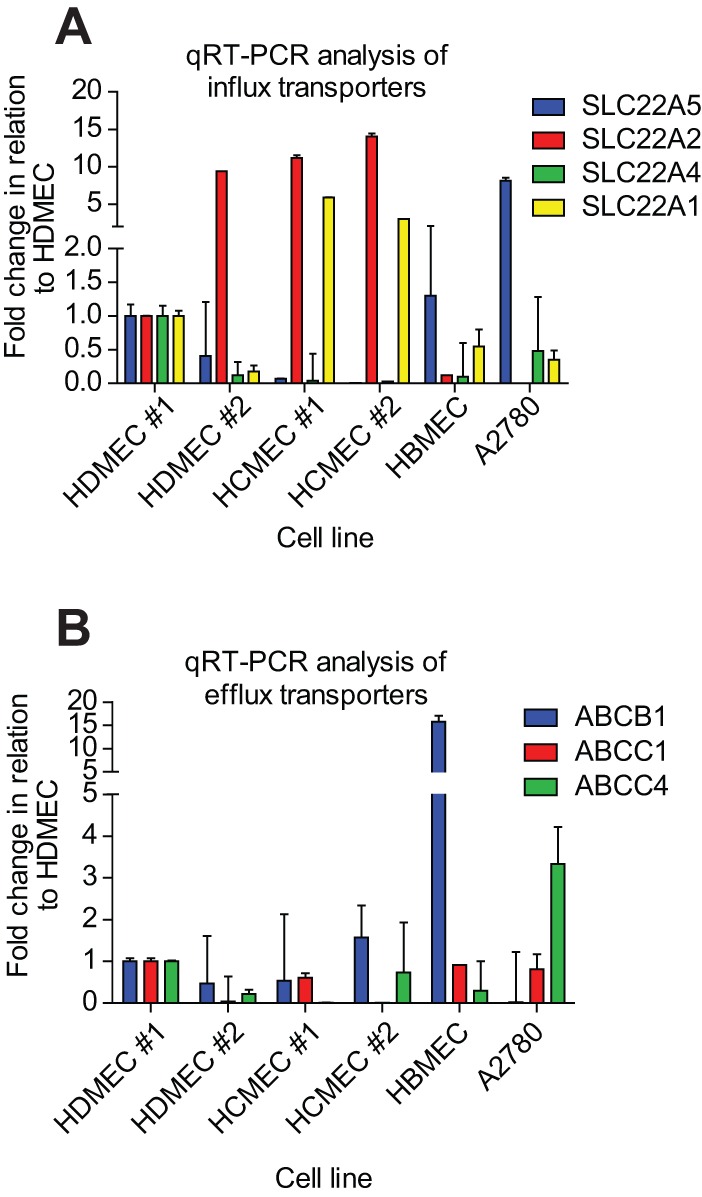
**Expression levels of drug influx and efflux transporters in different human cell lines.** Total RNA was isolated from HDMEC #1, HDMEC #2, HCMEC #1, HCMEC #2, HBMEC and A2780 cells and mRNA levels analysed by qRT-PCR for expression of: (A) organic cation influx transporters, (B) drug efflux transporters. (*n*=3), mean±s.d.

The ability of doxorubicin to affect permeability in HDMECs and HCMECs but not HBMECs suggests that endothelial cells in the BBB are resistant to the effect of doxorubicin, potentially due to decreased drug accumulation in these cells. We analysed the expression of different drug influx and efflux drug transporters across the range of endothelial cells. Anti-cancer drugs such as doxorubicin are thought to be transported into cells by organic cation transporters (Koepsell et al., 2007). We analysed mRNA expression of high affinity (OCTN1/SLC22A4, OCTN2/SLC22A5, OCT1/SLC22A1) and low affinity (OCT2/SLC22A2) organic cation transporters. The HCMECs express relatively low levels of the high-affinity OCTN1/SLC22A4 and OCTN2/SLC22A5 transporters and relatively high levels of the high-affinity OCT1/SLC22A1 and low-affinity OCT2/SLC22A2 transporters compared to the HBMECs and A2780 ovarian carcinoma cells (Fig. 2A).

**Fig. 3.**
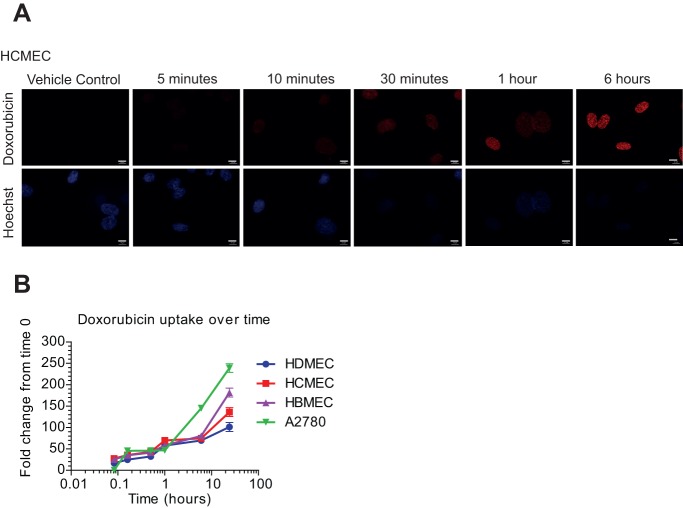
**Doxorubicin uptake and accumulation in cells.** (A) HCMEC were treated with 1 μM doxorubicin for time periods from 5 min to 6 h, cells were stained with Hoechst (blue) to visualise nuclei. Doxorubicin intrinsic fluorescence is detected at 568 nm, appearing in red. (B) Quantification of the levels of doxorubicin in HDMEC, HCMEC, HBMEC and A2780 over time (0-24 h) relative to protein concentration. Scale bars: 10 μm.

The drug efflux pump P-glycoprotein (P-gp, ABCB1/MDR-1) has been demonstrated to be involved in doxorubicin removal from cells (Sardi et al., 2013; Zhang et al., 2014) and has been shown to be expressed at high levels within the blood brain barrier (BBB) (Billson et al., 1994). HBMECs expressed relatively high levels of P-gp compared to the other endothelial cells (Fig. 2B) confirming their primary phenotype. Doxorubicin is intrinsically fluorescent (Karukstis et al., 1998), which allows for drug uptake and accumulation in cells to be monitored. We measured doxorubicin uptake over time both visually to determine intracellular localisation and quantitatively to determine how uptake varies between cell types. The data in Fig. 3A demonstrates that doxorubicin was taken up into HCMECs and accumulated in the nucleus over time. To assess the difference in accumulation within the different endothelial cells the fluorescence within the cells relative to the protein concentration was measured. A2780 cells showed the highest accumulation of doxorubicin over time; doxorubicin levels in all of the different endothelial cells were very similar (Fig. 3B). Taken together, this suggests that the differential effects of doxorubicin on tight junctions in HCMECs and HDMECs, compared with HBMECs, cannot be explained by potentially increased accumulation of doxorubicin in these cells.

### Analysis of molecular mechanism of Herceptin action on HCMECs

**Fig. 4.**
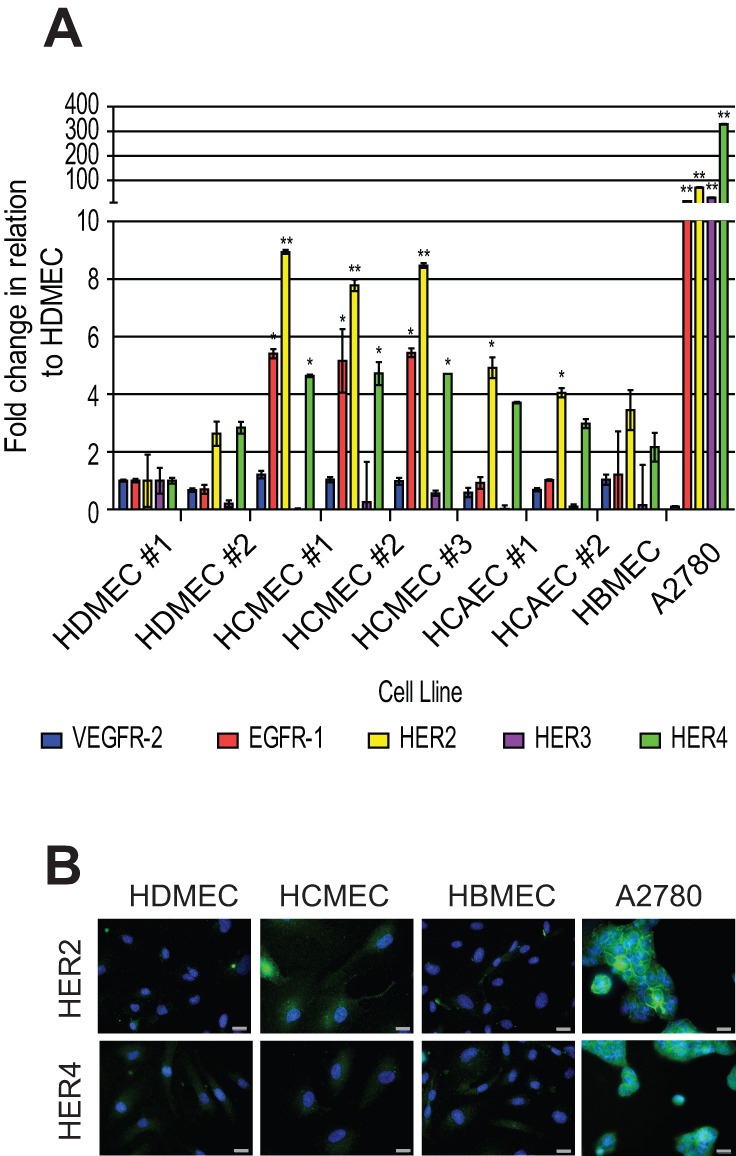
**Expression of EGF receptors in different human cell lines.** (A) Total RNA was extracted from cells and mRNA levels of *VEGFR-2*,* EGFR-1*,* HER2*,* HER3 *and* HER4* analysed by qRT-PCR, and compared to HDMEC #1. Mean±s.d. (*n*=3), *P≤0.05, one-way ANOVA, SPSS. (B) Immunofluorescence analysis of HER2 and HER4 levels (green) in HDMECs, HCMECs, HBMECs and A2780. Nuclei were visualised with Hoechst staining (blue). Scale bars: 10 μm. Results are from 1 experiment representative of 3.

Herceptin is known to target HER2 and inhibit the receptors ability to heterodimerise with HER3 and HER4 preventing activation and increasing endocytosis of HER2 (Yarden, 2001). Following the observation that Herceptin reduces tight junction formation and increases permeability in HCMECs, we investigated the expression of HER2 and HER4 in different endothelial cell types. We analysed a number of different batches of endothelial cells from different donors. We also utilised A2780 ovarian cancer cells, which are known to express HER2. Analysis of the expression levels of the EGFR family members *EGFR1*, *HER2*, *HER3* and *HER4* by qRT-PCR revealed that HCMECs express detectable levels of *EGFR-1* mRNA and *HER2* mRNA compared to other endothelial cells (Fig. 4A). This level of expression was considerably lower than the level expressed in the A2780 ovarian tumour cells. To confirm our findings, we analysed the expression of the HER2 protein by immunofluorescence, which revealed detectable levels of HER2 protein on the surface of the HCMECs relative to other endothelial cells (Fig. 4B).

**Fig. 5.**
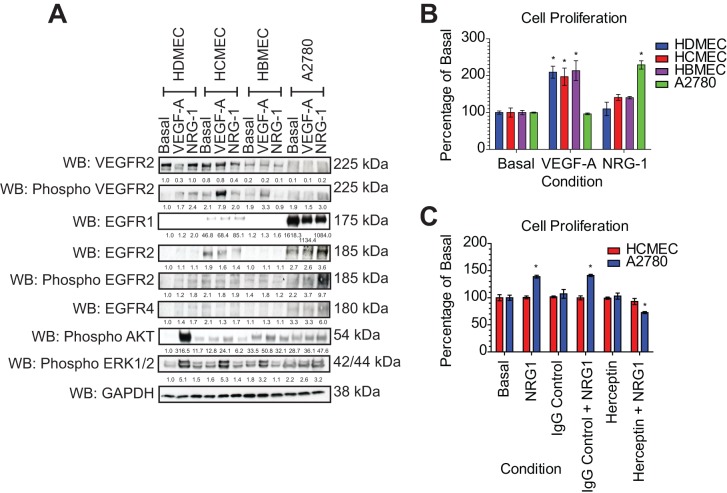
**Receptor tyrosine kinase activation in response to growth factor stimulation.** (A) HDMEC, HCMEC, HBMEC and A2780 were grown to sub-confluence and treated with either VEGF-A or NRG-1 at 50 ng/ml for 10 min. Intracellular signalling responses were analysed by western blotting for phosphorylation of VEGFR-2, HER2 (EGFR-2), AKT and ERK1/2 using phospho-specific antibodies. Total protein was measured for VEGFR-2, HER2 and HER4. Level of protein phosphorylation and protein levels are quantified relative to HDMEC basal level. (B) Cells were treated with 50 ng/ml VEGF-A or 50 ng/ml NRG-1 for 72 h. Cell proliferation was assessed by ATP Cell Titer Glo ATP assay. Data is presented as % of basal for each cell type, mean±s.d. (*n*=3), *P≤0.05 compared to basal, one-way ANOVA, SPSS. (C) Cells were treated with 50 ng/ml NRG-1 for 72 h following pre-incubation with human IgG control (10 µg/ml) or Herceptin (10 µg/ml). Data is presented as % of basal for each cell type, mean±s.d. (*n*=3), *P≤0.05, one-way ANOVA, SPSS.

The expression of HER2 on HCMECs may have a functional significance with respect to activation of intracellular signalling pathways. There is no known ligand for HER2, but the receptor is known to dimerise with HER3 and HER4. We used the ligand neuregulin-1 (NRG-1), which is known to bind and activate HER4 (Lemmens et al., 2006; Yarden, 2001). Stimulation with NRG-1 failed to evoke activation of HER2 and downstream activation of AKT and ERK1/2 in all endothelial cells (Fig. 5A). In contrast, VEGF-A, which binds to the VEGFR-2 present on endothelial cells (Holmes et al., 2007) was able to stimulate AKT phosphorylation and ERK1/2 phosphorylation in all endothelial cells. NRG-1 was able to stimulate HER2 phosphorylation in the A2780 ovarian cancer cells, in agreement with the expression of *HER3* and *HER4 mRNA* in these cells (Fig. 5A). Analysis of cell proliferation following ligand stimulation confirmed that whilst endothelial cells responded to VEGF-A, they did not respond to NRG-1. In contrast, NRG-1 was able to evoke a proliferative response in the A2780 cells (Fig. 5B). Taken together, these data suggest that whilst HCMECs express detectable levels of EGFR-1 and HER2, the lack of detectable levels of HER4 prevents dimerisation and ligand induced activation of HER2, and concomitant activation of downstream signalling pathways.

### Analysis of effect of doxorubicin and Herceptin on cell viability

**Fig. 6.**
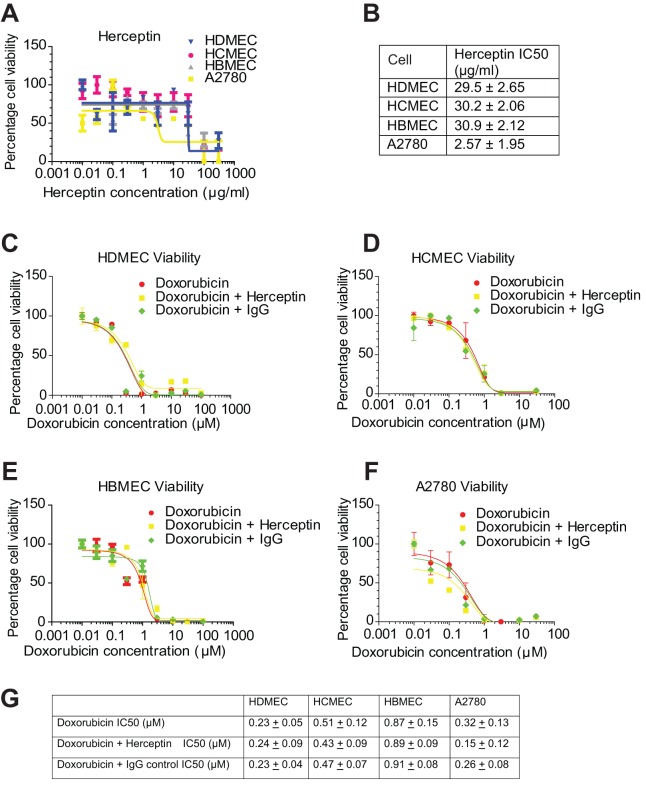
**Doxorubicin viability in the presence and absence of Herceptin.** (A) HDMEC, HCMEC, HBMEC and A2780 cells were incubated with Herceptin at a range of concentrations for 72 h and cell viability determined by Cell Titer Glo ATP assay. (B) Herceptin IC50s were generated. (C) HDMEC, (D) HCMEC, (E) HBMEC and (F) A2780 were treated with doxorubicin alone and in the presence of Herceptin (10 μg/ml) or a human IgG control (10 μg/ml). Cell viability was measured by Cell Titer Glo ATP assay. (G) IC_50_ levels were determined, mean±s.d. (*n*=4).

Herceptin is known to bind to HER2 expressed on breast and ovarian cancer cells causing cytotoxicity *in vivo* by inhibiting HER2-mediated intracellular signalling, increasing HER2 endocytosis and stimulating antibody-directed cell cytotoxicity (ADCC) (Collins et al., 2012; Kute et al., 2012; Yoshida et al., 2012). Addition of Herceptin alone caused a reduction in cell viability in the A2780 cells (Fig. 6A) with an IC_50_ of 2.6 μM; endothelial cells were not as sensitive to Herceptin with IC_50_s of approximately 30 μM. We were interested in determining the effect of co-addition of doxorubicin and Herceptin on cell viability *in vitro*. Addition of Herceptin caused a reduction in cell viability in the presence of doxorubicin, compared to doxorubicin alone, in the A2780 cells (Fig. 6F), with no enhancement of doxorubicin toxicity evident in the endothelial cells (Fig. 6C-E).

## DISCUSSION

Cardiotoxicity is a problem with current chemotherapy and has previously been thought to relate to adverse effects on cardiomyocytes (Adamcová et al., 2007; Lemmens et al., 2006). However, there is a growing awareness that non-cardiomyocyte cells such as endothelial cells can be adversely affected by cancer therapy (Wolf and Baynes, 2006). Our data shows that doxorubicin and Herceptin can increase cardiac microvascular endothelial cell permeability resulting in increased paracellular permeability. In patients, this would potentially lead to exposure of cardiomyocytes to doxorubicin and Herceptin leading to adverse effects of the drugs.

Anthracycline-induced cardiotoxicity has traditionally focused on effects on cardiac myocytes that lead to contractile dysfunction. Recent data has shown that doxorubicin can affect endothelial cells leading to increased intracellular redox stress (Wojcik et al., 2014) and apoptosis (Takemura and Fujiwara, 2007). Our data shows that doxorubicin can affect cardiac and dermal microvascular endothelial cell barrier function leading to loss of ZO-1 in tight junctions and an increase in paracellular permeability (Fig. 1A-E). This effect was evident after 3-6 h and occurred in the absence of apoptosis, suggesting that it is not a consequence of programmed cell death. The inability of doxorubicin to affect brain microvascular endothelial cell tight junction formation and permeability was in agreement with the fact that *in vivo* doxorubicin does not cross the BBB, precluding its use in the treatment of brain malignancies (Deeken and Loscher, 2007).

Doxorubicin is thought to be transported into cells by the organic cation transporters (OCT). Interestingly, the HCMECs expressed relatively high levels of the recently identified high affinity doxorubicin transporter OCT1/SLC22A1 (Andreev et al., 2016). However, the differential effects of doxorubicin on the endothelial cells were not due to differences in drug accumulation as doxorubicin accumulated to similar levels over 8 h in all endothelial cells, with higher levels evident in the A2780 ovarian carcinoma cells.

The ability of doxorubicin to affect cardiac microvascular endothelial cell permeability *in vivo* has been reported in rat studies where cardiac permeability changes correlated with decreased left ventricular function (Fernandez-Fernandez et al., 2014). Taken together, our data suggests that doxorubicin can interfere with tight junction formation between cardiac microvascular endothelial cells leading to increased permeability to the drug and ultimately detrimental effects on the underlying myocardium.

Anthracyclines are often utilised with Herceptin in drug treatment regimens for breast and ovarian cancers overexpressing HER2 (Pegram et al., 1999). Concurrent treatment with these drugs leads to increased cardiotoxicity through significantly decreased LVEF which has led to the sequential use of these drugs in an attempt to minimise cardiotoxicity. The ability of Herceptin to adversely affect cardiac function has been attributed to binding to HER2 receptors on cardiomyocytes leading to ‘on-target’ toxicity. The importance of HER2 in heart development has been linked to expression of the receptor in myocytes (Harari and Yarden, 2000). Endothelial cells are known to secrete neuregulins which can stimulate EGFRs on myocytes in a paracrine manner (Lemmens et al., 2006), regulating heart development (Lee et al., 1995). The reduced formation of the ventricular myocardial trabecular in *Her2* null mice produces a lethal phenotype at E9.5-10.5 (Harari and Yarden, 2000). During morphogenesis of the heart the myocardial trabecular functions to supply a blood flow. The reduced formation of the myocardial trabecular consequently results in reduced blood flow, which is considered a probable explanation for lethality in *Her2* null mice (Lee et al., 1995). Although the reduced blood flow could be due to endothelial defects, HER2 function has not, to our knowledge, been investigated for its role in endothelial cell function.

Our data shows that Herceptin is able to reduce cardiac microvascular tight junction formation resulting in a concomitant increase in permeability (Fig. 1B,E), an effect not observed in the other endothelial cells. This effect was also increased in the presence of doxorubicin suggesting that cardiac microvascular endothelial barrier formation was uniquely sensitive to a combination of both drugs. Analysis of the expression levels of EGFR family members revealed that HCMECs express significantly higher levels of EGFR-1, HER2 and HER4 than dermal or brain microvascular endothelial cells; however, the HER2 did not appear to be functional as neuregulin did not evoke HER2 phosphorylation and a subsequent intracellular signalling response (Fig. 5A) and proliferation (Fig. 5B) in the HCMECs in contrast to the A2780 ovarian cancer cells (Fig. 5A-C). This is most probably due to the fact that expression of HER2 and HER3 in the HCMECs is an order of magnitude lower than in the A2780 cells preventing receptor dimerization in the presence of the NRG-1 ligand (Fig. 4). However, whilst the HER2 expression in HCMECs was not high enough to facilitate a NRG-1 response, it was high enough to be detected by immunofluorescence with an anti-HER2 antibody. This data suggests that Herceptin can bind to HER2 on the surface of HCMECs and potentially interfere with tight junction formation. A link between HER2 and ZO-1 has been defined in endometrial Ishikawa cells, where downregulation of HER2 led to arecoline-induced alterations in tight junctions (Giri et al., 2010).

Herceptin binding to HER2 *in vivo* is able to prevent cancer cell proliferation by three defined pathways: inhibition of intracellular signalling, sequestration of the immune system, and receptor internalisation (Kute et al., 2009). Analysis of Herceptin effects on cell viability revealed that the endothelial cells were relatively insensitive to this drug compared with the A2780 cells (Fig. 6A,B). Furthermore, an additive effect on cell viability of co-addition of Herceptin and doxorubicin compared with doxorubicin alone was only evident in the A2780 cells confirming the relative insensitivity of endothelial cells to effects on cell viability with Herceptin.

Our data shows that the cardiotoxic drugs doxorubicin and Herceptin can both affect cardiac microvascular endothelial cell barrier function leading to increased permeability. This suggests that the cardiotoxicity observed *in vivo* with these agents may be due to multi-cellular effects with adverse effects on the cardiac endothelium a potentially initiating and contributory event in drug-induced cardiotoxicity.

## MATERIALS AND METHODS

### Cell culture

Human dermal microvascular endothelial cells (HDMECs, #C-12212, lot-0092101.2 and 6060707.1), human cardiac microvascular endothelial cells (HCMECs, #C12285, lot-3011401, 9090701.2 and 1122702) and human brain microvascular endothelial cells (HBMECs, #C-12287, lot-1111603.7) were purchased from PromoCell (Heidelberg, Germany). Endothelial cells were routinely cultured on 0.5% (w/v) gelatin-coated cell culture dishes (G1890, Sigma, Poole, UK) at passages 2-10. Endothelial cells were cultured in endothelial basal MV2 medium (EBM) supplemented with 5% v/v fetal calf serum (FCS), recombinant epidermal growth factor (EGF, 5 ng/ml), recombinant human basic fibroblast growth factor (FGF, 10 ng/ml), long R3 insulin-like growth factor (IGF, 20 ng/ml), recombinant human vascular endothelial growth factor-A_165_ (VEGF-A, 0.5 ng/ml), ascorbic acid (1 µg/ml) and hydrocortisone (0.2 µg/ml), referred to herein as endothelial full growth-medium (FGM). A2780 were routinely cultured in RPMI 1640 medium (#61870044, Gibco) supplemented with 10% v/v FCS. All cells were incubated at 37°C in humidified air containing 5% (v/v) CO_2_.

### Cell stimulation and preparation of cell lysates

Before stimulation with agonists, cells were serum-starved for 20 h in EBM supplemented with 1% v/v FCS for HDMECs, HCMECs and HBMECs or RPMI 1640 supplemented with 1% FCS for A2780. Cells were stimulated with 50 ng/ml agonists (VEGF-A, NRG-1; Peprotech, Rocky Hill, NJ) for 10 min. Cells were washed in ice-cold Dulbecco's phosphate-buffered saline (DPBS, Lonza, Basel, Switzerland) and lysed on ice in modified RIPA buffer (20 mM Tris pH 7.5, 150 mM NaCl, 2.5 mM EDTA, 10% w/v glycerol, 1% v/v Triton X-100, 1 mM Na_3_VO_4_, 10 µg/ml aprotinin, leupeptin and pepstatin A, 1 mM PMSF, 0.5% v/v SDS and 0.5% v/v sodium deoxycholate). Lysates were centrifuged [17,000 ***g*** (14,000 RPM) for 20 min at 4°C] before diluting in LDS sample buffer (Invitrogen, Paisley, UK) containing 2.5% (v/v) 2-mercaptoethanol (Sigma, Poole, UK) and denaturing at 90°C for 6 min.

### Western blot analysis

Proteins were resolved by SDS-PAGE on 10% tris-glycine gels and transferred to 0.2 µm nitrocellulose membrane (Hybond-C, GE Healthcare, Amersham, UK). Membranes were then blocked in 5% w/v bovine serum albumin (BSA) in Tris-buffered saline (TBS) with 0.1% v/v Tween-20 (TBST). Membranes were probed overnight with primary antibodies diluted in 2% w/v BSA in TBST, directed against: phosphorylated AKT (Ser473), phosphorylated ERK1/2 (Thr202/Tyr204), glyceraldehyde 3-phosphate dehydrogenase (GAPDH), caspase 3, cleaved caspase 3 (Asp175), HER2 and HER4 (New England Biolabs, Hitchin, UK). Antibody to ZO-1 (#61-7300) was purchased from Invitrogen (Thermo Fisher Scientific) and CD31 (#M082329-2) purchased from Dako (Denmark). Proteins were detected by using rabbit- or mouse-specific HRP secondary antibodies (Jackson Labs) and enhanced-chemiluminescence (ECL) western blotting detection reagent (Pierce).

### Quantitative real-time PCR (qRT-PCR) analysis

Total RNA was extracted from cells using the RNAeasy kit following the manufacturer's instructions (Qiagen, Crawley, UK). Synthesis of cDNA was conducted by reverse transcription using 1 µg total RNA, oligo dT_18_, RNase OUT, M-MLV Reverse Transcriptase (Invitrogen, Thermo Fisher Scientific). Mixtures used for qRT-PCR included: 1.5 µl cDNA, 3 µl dH_2_O (RNase and DNase free), 12.5 µl 2X Power SYBR^®^ Green mastermix (Applied Biosystems, Warrington, UK) and 0.25 µM forward and reverse primers (Invitrogen). Primers: *EGFR1* forward 5′ TAT GTT CCC TCC AGG TCA GC 3′; *EGFR1* reverse 5′ GCA CCT GTA AAA TGC CCT GT 3′; *HER2* forward 5′ CTA CGG CAG AGA ACC CAG AG 3′; *HER2* reverse 5′ CTT GAT GCC AGC AGA AGT CA 3′; *HER3* forward 5′ CTT ATC CGA GGG CAA ATT CA 3′; *HER3* reverse 5′ TTT CCC TTA GTT CCC CAT CC 3′; *HER4* forward 5′ TGT GTT CCA GTG ATG GCT GT 3′; *HER4* reverse 5′ CCA TTC TCA AAC TCC CGA AA 3′; *P-gp* forward 5′ GTG GGG CAA GTC AGT TCA TT 3′; *P-gp* reverse 5′ TTC CAA TGT GTT CGG CAT TA 3′; *ABCC1* forward 5′ GCC GGT GAA GGT TGT GTA CT 3′; *ABCC1* reverse 5′ AGG GGT TCC ACT CCT TCT GT 3′; *ABCC4* forward 5′ GGC GAA TTG TTA GCT GTG GT 3′; *ABCC4* reverse 5′ CAG GGC TGC TGA GAC ACA TA 3′; *SLC22A5* forward 5′ CTG GTG GTT CAT CCC TGA GT 3′; *SLC22A5* reverse 5′ AGT GGA AGG CAC AAC AAT CC 3′; *SLC22A2* forward 5′ TCG CTT AAT CCA AGG ACT GG 3′; *SLC22A2* reverse 5′ CAC CAG GAG CCC AAC TGT AT 3′; *SLC22A4* forward 5′ CTG CCC AGG CGT TAT ATC AT 3′; *SLC22A4* reverse 5′ AAT TTT CCC AGC ATG ACC AG 3′. qRT-PCR reactions were performed on a ViiA7 Real-Time PCR system (Applied Biosystems, Foster City, CA) using the parameters: 50°C for 2 min and 95°C for 10 min followed by 95°C for 15 s and 60°C for 1 min, repeating for 40 cycles. Cycle threshold (C_T_) values were obtained for the mRNA of interest to compare to the GAPDH control to determine gene expression changes using the Comparative C_T_ (2^−ΔΔCT^) method (Livak and Schmittgen, 2001).

### Doxorubicin and Herceptin treatment of cells

HDMECs, HCMECs and HBMECs were plated on gelatin-coated 13 mm glass coverslips and placed in the well of a 24-well plate and allowed to reach confluence over a 7 day period with media changes every 2-3 days. Cells were treated with doxorubicin (purchased from Sigma, 30 mM stock in DMSO) diluted to 0.1 µM in endothelial FGM to ensure final 0.1% v/v DMSO concentration. Herceptin (purchased from Clatterbridge Hospital Pharmacy, 100 mg/ml stock in PBS) was used at 10 µg/ml diluted in endothelial FGM. Control human IgG (#31154) was purchased from Invitrogen (Thermo Fisher Scientific). Drugs were added to the cells individually or in combination for 6 h.

### Immunofluorescence

HDMECs, HCMECs, HBMECs and A2780 grown on glass coverslips were fixed in 2% w/v paraformaldehyde (PFA), permeabilised in 0.25% (v/v) Triton X-100, blocked in 1% BSA in TBST with 5% v/v donkey serum for 1 h. Samples were incubated with primary antibodies for 1 h: HER2 and HER4 (New England Biolabs, Hitchin, UK) and ZO-1 (ThermoFisher). Cells were washed and incubated with Alexa Fluor^®^ secondary antibodies donkey-anti-rabbit IgG (H+L) 488 (Invitrogen, Thermo Fisher Scientific) for 1 h. Additional to ZO-1 cells were stained with Alexa Fluor^®^ Phalloidin 568 incubated additional to secondary antibody. Nuclei were labelled with 10 μg/ml Hoechst 33342 (Invitrogen, Thermo Fisher Scientific) for 10 min. Coverslips were mounted using Prolong Gold mounting medium (Invitrogen, Thermo Fisher Scientific) and visualised on a Zeiss AxioObserver Z1 inverted fluorescence microscope with Apotome2 optical sectioning device.

### Permeability

HDMECs, HCMECs and HBMECs were grown to confluence over 7 days on ThinCerts™ 0.4 µm translucent (Greiner). Cells were treated with drugs for 6 h before washing the inserts in DPBS and addition of 4 kDa FITC fluorescent dextran (Sigma) diluted in Phenol Red free endothelial FGM for 25 min. The flow through was measured by taking a sample of medium from the surrounding well and measuring the level of fluorescence (Varioskan plate reader at Ex 490 nm and Em 525 nm).

### Cell viability

HDMECs, HCMECs, HBMECs and A2780 were grown to sub-confluence in gelatin-coated 96-well plates before being treated with doxorubicin (100 µM – 0.03 µM) alone or in combination with Herceptin 10 µg/ml or IgG control 10 µg/ml for 72 h. Cells were lysed in Cell Titer Glo (Promega) and the contents transferred to white 96-well flat bottomed plates and the bioluminescence measured (Varioskan plate reader).

### Statistical significance

This was performed using a one-way analysis of variance (ANOVA) followed by Dunnett's *post hoc* test using SPSS software. Significant differences between control and test groups were evaluated with *P values <0.05, indicated on the graphs. Error bars in graphs and histograms denote ±s.d. (standard deviation).

## References

[BIO020362C1] Adamcová M., Šimůnek T., Kaiserová H., Popelová O., Štěrba M., Potáčová A., Vávrová J., Maláková J. and Geršl V. (2007). In vitro and in vivo examination of cardiac troponins as biochemical markers of drug-induced cardiotoxicity. *Toxicology* 237, 218-228. 10.1016/j.tox.2007.05.016 17587482

[BIO020362C2] Andreev E., Brosseau N., Carmona E., Mes-Masson A.-M. and Ramotar D. (2016). The human organic cation transporter OCT1 mediates high affinity uptake of the anticancer drug daunorubicin. *Sci. Rep.* 6, 20508 10.1038/srep20508 26861753 PMC4748219

[BIO020362C3] Baselga J., Norton L., Albanell J., Kim Y. M. and Mendelsohn J. (1998). Recombinant humanized anti-HER2 antibody (Herceptin) enhances the antitumor activity of paclitaxel and doxorubicin against HER2/neu overexpressing human breast cancer xenografts. *Cancer Res.* 58, 2825-2831. 9661897

[BIO020362C4] Bazzoni G. and Dejana E. (2004). Endothelial cell-to-cell junctions: molecular organization and role in vascular homeostasis. *Physiol. Rev.* 84, 869-901. 10.1152/physrev.00035.2003 15269339

[BIO020362C5] Billingham M. E., Mason J. W., Bristow M. R. and Daniels J. R. (1978). Anthracycline cardiomyopathy monitored by morphologic changes. *Cancer Treat. Rep.* 62, 865-872. 667860

[BIO020362C6] Billson A. L., Palmer J. B., Walker D. A. and Lowe J. (1994). Multidrug resistance gene (MDR 1) expression in neuro-axial tumours of children and young adults. *Br. J. Neurosurg.* 8, 585-591. 10.3109/02688699409002952 7857540

[BIO020362C7] Blasberg R. G. and Groothuis D. R. (1986). Chemotherapy of brain tumors: physiological and pharmacokinetic considerations. *Semin. Oncol.* 13, 70-82. 3513317

[BIO020362C8] Brutsaert D. L. (2003). Cardiac endothelial-myocardial signaling: its role in cardiac growth, contractile performance, and rhythmicity. *Physiol. Rev.* 83, 59-115. 10.1152/physrev.00017.2002 12506127

[BIO020362C9] Chintalgattu V., Rees M. L., Culver J. C., Goel A., Jiffar T., Zhang J., Dunner K. Jr., Pati S., Bankson J. A., Pasqualini R. et al. (2013). Coronary microvascular pericytes are the cellular target of sunitinib malate-induced cardiotoxicity. *Sci. Transl. Med.* 5, 187ra69 10.1126/scitranslmed.3005066 PMC3833098 23720580

[BIO020362C10] Chiusa M., Hool S.-L., Truetsch P., Djafarzadeh S., Jakob S. M., Seifriz F., Scherer S. J., Suter T. M., Zuppinger C. and Zbinden S. (2012). Cancer therapy modulates VEGF signaling and viability in adult rat cardiac microvascular endothelial cells and cardiomyocytes. *J. Mol. Cell Cardiol.* 52, 1164-1175. 10.1016/j.yjmcc.2012.01.022 22326847

[BIO020362C11] Chung A. S., Lee J. and Ferrara N. (2010). Targeting the tumour vasculature: insights from physiological angiogenesis. *Nat. Rev. Cancer* 10, 505-514. 10.1038/nrc2868 20574450

[BIO020362C12] Collins D. M., O'Donovan N., McGowan P. M., O'Sullivan F., Duffy M. J. and Crown J. (2012). Trastuzumab induces antibody-dependent cell-mediated cytotoxicity (ADCC) in HER-2-non-amplified breast cancer cell lines. *Ann. Oncol.* 23, 1788-1795. 10.1093/annonc/mdr484 22056974

[BIO020362C13] Creedon H., Byron A., Main J., Hayward L., Klinowska T. and Brunton V. G. (2014). Exploring mechanisms of acquired resistance to HER2 (human epidermal growth factor receptor 2)-targeted therapies in breast cancer. *Biochem. Soc. Trans.* 42, 822-830. 10.1042/BST20140109 25109964

[BIO020362C14] Cross M. J., Berridge B. R., Clements P. J., Cove-Smith L., Force T. L., Hoffmann P., Holbrook M., Lyon A. R., Mellor H. R., Norris A. A. et al. (2015). Physiological, pharmacological and toxicological considerations of drug-induced structural cardiac injury. *Br. J. Pharmacol.* 172, 957-974. 10.1111/bph.12979 25302413 PMC4314188

[BIO020362C15] Deeken J. F. and Loscher W. (2007). The blood-brain barrier and cancer: transporters, treatment, and Trojan horses. *Clin. Cancer Res.* 13, 1663-1674. 10.1158/1078-0432.CCR-06-2854 17363519

[BIO020362C16] Ewer M. S. and Lippman S. M. (2005). Type II chemotherapy-related cardiac dysfunction: time to recognize a new entity. *J. Clin. Oncol.* 23, 2900-2902. 10.1200/JCO.2005.05.827 15860848

[BIO020362C17] Fernandez-Fernandez A., Carvajal D. A., Lei T. and McGoron A. J. (2014). Chemotherapy-induced changes in cardiac capillary permeability measured by fluorescent multiple indicator dilution. *Ann. Biomed. Eng.* 42, 2405-2415. 10.1007/s10439-014-1110-9 25224075 PMC4241122

[BIO020362C18] Force T., Krause D. S. and Van Etten R. A. (2007). Molecular mechanisms of cardiotoxicity of tyrosine kinase inhibition. *Nat. Rev. Cancer* 7, 332-344. 10.1038/nrc2106 17457301

[BIO020362C19] Gianni L., Salvatorelli E. and Minotti G. (2007). Anthracycline cardiotoxicity in breast cancer patients: synergism with trastuzumab and taxanes. *Cardiovasc. Toxicol.* 7, 67-71. 10.1007/s12012-007-0013-5 17652806

[BIO020362C20] Giri S., Poindexter K. M., Sundar S. N. and Firestone G. L. (2010). Arecoline induced disruption of expression and localization of the tight junctional protein ZO-1 is dependent on the HER 2 expression in human endometrial Ishikawa cells. *BMC Cell Biol.* 11, 53 10.1186/1471-2121-11-53 20604955 PMC2910664

[BIO020362C21] González-Mariscal L., Nava P. and Hernandez S. (2005). Critical role of tight junctions in drug delivery across epithelial and endothelial cell layers. *J. Membr. Biol.* 207, 55-68. 10.1007/s00232-005-0807-y 16477528

[BIO020362C22] González-Mariscal L., Tapia R. and Chamorro D. (2008). Crosstalk of tight junction components with signaling pathways. *Biochim. Biophys. Acta* 1778, 729-756. 10.1016/j.bbamem.2007.08.018 17950242

[BIO020362C23] Harari D. and Yarden Y. (2000). Molecular mechanisms underlying ErbB2/HER2 action in breast cancer. *Oncogene* 19, 6102-6114. 10.1038/sj.onc.1203973 11156523

[BIO020362C24] Holmes K., Roberts O. L., Thomas A. M. and Cross M. J. (2007). Vascular endothelial growth factor receptor-2: structure, function, intracellular signalling and therapeutic inhibition. *Cell Signal.* 19, 2003-2012. 10.1016/j.cellsig.2007.05.013 17658244

[BIO020362C25] Karukstis K. K., Thompson E. H. Z., Whiles J. A. and Rosenfeld R. J. (1998). Deciphering the fluorescence signature of daunomycin and doxorubicin. *Biophys. Chem.* 73, 249-263. 10.1016/S0301-4622(98)00150-1 9700924

[BIO020362C26] Koepsell H., Lips K. and Volk C. (2007). Polyspecific organic cation transporters: structure, function, physiological roles, and biopharmaceutical implications. *Pharm. Res.* 24, 1227-1251. 10.1007/s11095-007-9254-z 17473959

[BIO020362C27] Kute T. E., Savage L., Stehle J. R. Jr, Kim-Shapiro J. W., Blanks M. J., Wood J. and Vaughn J. P. (2009). Breast tumor cells isolated from in vitro resistance to trastuzumab remain sensitive to trastuzumab anti-tumor effects in vivo and to ADCC killing. *Cancer Immunol. Immunother.* 58, 1887-1896. 10.1007/s00262-009-0700-0 19340424 PMC11030142

[BIO020362C28] Kute T., Stehle J. R. Jr, Ornelles D., Walker N., Delbono O. and Vaughn J. P. (2012). Understanding key assay parameters that affect measurements of trastuzumab-mediated ADCC against Her2 positive breast cancer cells. *Oncoimmunology* 1, 810-821. 10.4161/onci.20447 23162748 PMC3489736

[BIO020362C29] Laverty H., Benson C., Cartwright E., Cross M., Garland C., Hammond T., Holloway C., McMahon N., Milligan J., Park B. et al. (2011). How can we improve our understanding of cardiovascular safety liabilities to develop safer medicines? *Br. J. Pharmacol.* 163, 675-693. 10.1111/j.1476-5381.2011.01255.x 21306581 PMC3111672

[BIO020362C30] Lee K.-F., Simon H., Chen H., Bates B., Hung M.-C. and Hauser C. (1995). Requirement for neuregulin receptor Erbb2 in neural and cardiac development. *Nature* 378, 394-398. 10.1038/378394a0 7477377

[BIO020362C31] Lemmens K., Segers V. F. M., Demolder M. and De Keulenaer G. W. (2006). Role of neuregulin-1/ErbB2 signaling in endothelium-cardiomyocyte cross-talk. *J. Biol. Chem.* 281, 19469-19477. 10.1074/jbc.M600399200 16698793

[BIO020362C32] Lemmens K., Doggen K. and De Keulenaer G. W. (2007). Role of neuregulin-1/ErbB signaling in cardiovascular physiology and disease: implications for therapy of heart failure. *Circulation* 116, 954-960. 10.1161/CIRCULATIONAHA.107.690487 17709650

[BIO020362C33] Livak K. J. and Schmittgen T. D. (2001). Analysis of relative gene expression data using real-time quantitative PCR and the 2−ΔΔCT Method. *Methods* 25, 402-408. 10.1006/meth.2001.1262 11846609

[BIO020362C34] Mikaelian I., Buness A., de Vera-Mudry M.-C., Kanwal C., Coluccio D., Rasmussen E., Char H. W., Carvajal V., Hilton H., Funk J. et al. (2010). Primary endothelial damage is the mechanism of cardiotoxicity of tubulin-binding drugs. *Toxicol. Sci.* 117, 144-151. 10.1093/toxsci/kfq189 20624997

[BIO020362C35] Pegram M., Hsu S., Lewis G., Pietras R., Beryt M., Sliwkowski M., Coombs D., Baly D., Kabbinavar F. and Slamon D. (1999). Inhibitory effects of combinations of HER-2/neu antibody and chemotherapeutic agents used for treatment of human breast cancers. *Oncogene* 18, 2241-2251. 10.1038/sj.onc.1202526 10327070

[BIO020362C36] Porter K. E. and Turner N. A. (2009). Cardiac fibroblasts: at the heart of myocardial remodeling. *Pharmacol. Ther.* 123, 255-278. 10.1016/j.pharmthera.2009.05.002 19460403

[BIO020362C37] Privratsky J. R., Paddock C. M., Florey O., Newman D. K., Muller W. A. and Newman P. J. (2011). Relative contribution of PECAM-1 adhesion and signaling to the maintenance of vascular integrity. *J. Cell Sci.* 124, 1477-1485. 10.1242/jcs.082271 21486942 PMC3078814

[BIO020362C38] Ravenscroft S. M., Pointon A., Williams A. W., Cross M. J. and Sidaway J. E. (2016). Cardiac non-myocyte cells show enhanced pharmacological function suggestive of contractile maturity in stem cell derived cardiomyocyte microtissues. *Toxicol. Sci.* 152, 99-112. 10.1093/toxsci/kfw069 27125969 PMC4922542

[BIO020362C39] Sandoo A., Kitas G. D. and Carmichael A. R. (2014). Endothelial dysfunction as a determinant of trastuzumab-mediated cardiotoxicity in patients with breast cancer. *Anticancer Res.* 34, 1147-1151. 24596352

[BIO020362C40] Sardi I., la Marca G., Cardellicchio S., Giunti L., Malvagia S., Genitori L., Massimino M., de Martino M. and Giovannini M. G. (2013). Pharmacological modulation of blood-brain barrier increases permeability of doxorubicin into the rat brain. *Am. J. Cancer Res.* 3, 424-432. 23977451 PMC3744021

[BIO020362C41] Sartiano G. P., Lynch W. E. and Bullington W. D. (1979). Mechanism of action of the anthracycline anti-tumor antibiotics, doxorubicin, daunomycin and rubidazone: preferential inhibition of DNA polymerase alpha. *J. Antibiot.* 32, 1038-1045. 10.7164/antibiotics.32.1038 528363

[BIO020362C42] Takemura G. and Fujiwara H. (2007). Doxorubicin-induced cardiomyopathy. *Prog. Cardiovasc. Dis.* 49, 330-352. 10.1016/j.pcad.2006.10.002 17329180

[BIO020362C43] Tirziu D., Giordano F. J. and Simons M. (2010). Cell communications in the heart. *Circulation* 122, 928-937. 10.1161/CIRCULATIONAHA.108.847731 20805439 PMC2941440

[BIO020362C44] Walshe J. M., Denduluri N., Berman A. W., Rosing D. R. and Swain S. M. (2006). A phase II trial with trastuzumab and pertuzumab in patients with HER2-overexpressed locally advanced and metastatic breast cancer. *Clin. Breast Cancer* 6, 535-539. 10.3816/CBC.2006.n.009 16595039

[BIO020362C45] Wojcik T., Buczek E., Majzner K., Kolodziejczyk A., Miszczyk J., Kwiatek W., Baranska M., Szymonski M. and Chlopicki S. (2014). Comparative endothelial profiling of doxorubicin and daunorubicin in cultured endothelial cells. *Toxicol In Vitro* 3, 512-521. 10.1016/j.tiv.2014.12.009 25529801

[BIO020362C46] Wolf M. B. and Baynes J. W. (2006). The anti-cancer drug, doxorubicin, causes oxidant stress-induced endothelial dysfunction. *Biochim. Biophys. Acta* 1760, 267-271. 10.1016/j.bbagen.2005.10.012 16337743

[BIO020362C47] Yarden Y. (2001). The EGFR family and its ligands in human cancer. signalling mechanisms and therapeutic opportunities. *Eur. J. Cancer* 37 Suppl. 4, S3-S8. 10.1016/s0959-8049(01)00230-1 11597398

[BIO020362C48] Yoshida R., Tazawa H., Hashimoto Y., Yano S., Onishi T., Sasaki T., Shirakawa Y., Kishimoto H., Uno F., Nishizaki M. et al. (2012). Mechanism of resistance to trastuzumab and molecular sensitization via ADCC activation by exogenous expression of HER2-extracellular domain in human cancer cells. *Cancer Immunol. Immunother.* 61, 1905-1916. 10.1007/s00262-012-1249-x 22465967 PMC11028791

[BIO020362C49] Zhang J., Sun T., Liang L., Wu T. and Wang Q. (2014). Drug promiscuity of P-glycoprotein and its mechanism of interaction with paclitaxel and doxorubicin. *Soft Matter* 10, 438-445. 10.1039/C3SM52499J 24652302

